# Immersive training of clinical decision making with AI driven virtual patients – a new VR platform called medical tr.AI.ning

**DOI:** 10.3205/zma001600

**Published:** 2023-04-17

**Authors:** Marvin Mergen, Anna Junga, Benjamin Risse, Dimitar Valkov, Norbert Graf, Bernhard Marschall, medical tr.AI.ning consortium

**Affiliations:** 1 Saarland University, Department of Pediatric Oncology and Hematology, Homburg, Germany; 2 University of Münster, Institute of Education and Student Affairs, Münster, Germany; 3 Stiftungsklinikum PROSELIS, Department of Urology, Recklinghausen, Germany; 4 University of Münster, Institute for Geoinformatics, Münster, Germany; 5 University of Münster, Computer Vision & Machine Learning Systems Group, Münster, Germany; 6 Saarland University, Department of Computer Science, Homburg, Germany; 7 German Research Center for Artificial Intelligence (DFKI), Saarbrücken, Germany

**Keywords:** virtual reality, medical education, artificial intelligence, virtual patients

## Abstract

**Background:**

Medical students need to be prepared for various situations in clinical decision-making that cannot be systematically trained with real patients without risking their health or integrity.

To target system-related limitations of actor-based training, digital learning methods are increasingly used in medical education, with virtual reality (VR)- training seeming to have high potential. Virtually generated training scenarios allow repetitive training of highly relevant clinical skills within a protected, realistic learning environment.

Thanks to Artificial Intelligence (AI), face-to-face interaction with virtual agents is feasible. Combining this technology with VR-simulations offers a new way of situated context-based, first-person training for medical students.

**Project goal and method:**

The authors’ aim is to develop a modular digital training platform for medical education with virtual, interactable agents and to integrate this platform into the medical curriculum. The medical tr.AI.ning platform will provide veridical simulation of clinical scenarios with virtual patients, augmented with highly realistic medical pathologies within a customizable, realistic situational context.

Medical tr.AI.ning is scaled to four complementary developmental steps with different scenarios that can be used separately and so each outcome can successively be integrated early within the project. Every step has its own focus (visual, movement, communication, combination) and extends an author toolbox through its modularity. The modules of each step will be specified and designed together with medical didactics experts.

**Perspective:**

To ensure constant improvement of user experience, realism, and medical validity, the authors will perform regular iterative evaluation rounds.

Furthermore, integration of medical tr.AI.ning into the medical curriculum will enable long-term and large-scale detection of benefits and limitations of this approach, providing enhanced alternative teaching paradigms for VR technology.

## Zusammenfassung

**Hintergrund:**

Medizinstudierende müssen auf verschiedene Situationen in klinischer Entscheidungsfindung vorbereitet werden, die nicht systematisch mit echten Patienten geübt werden können, ohne ihre Gesundheit oder Integrität zu gefährden.

Um die systembedingten Einschränkungen des schauspielbasierten Trainings auszugleichen, werden in der medizinischen Ausbildung zunehmend digitale Lernmethoden eingesetzt, wobei Training in Virtueller Realität (VR) ein hohes Potenzial zu haben scheint. Virtuell generierte Trainingsszenarien ermöglichen das wiederholte Training hochrelevanter klinischer Fertigkeiten in einer geschützten, realistischen Lernumgebung.

Dank Künstlicher Intelligenz (KI) ist eine direkte Interaktion mit virtuellen Agenten möglich. Die Kombination dieser Technologie mit VR-Simulationen bietet eine neue Möglichkeit für ein kontextbezogenes, persönliches Training für Medizinstudierende.

**Projektziel und Methode:**

Das Ziel der Autoren ist es, eine modulare digitale Trainingsplattform für die medizinische Ausbildung mit virtuellen, interaktiven Agenten zu entwickeln und diese Plattform in das medizinische Curriculum zu integrieren. Die medical tr.AI.ning - Plattform wird eine realitätsnahe Simulation von klinischen Szenarien mit virtuellen Patienten ermöglichen, ergänzt durch hochrealistische medizinische Pathologien innerhalb eines anpassbaren, realistischen situativen Kontextes.

Medical tr.AI.ning ist auf vier komplementäre Entwicklungsschritte mit unterschiedlichen Szenarien skaliert, die separat genutzt werden können, so dass jedes Ergebnis sukzessive früh in das Projekt integriert werden kann. Jeder Schritt hat seinen eigenen Schwerpunkt (Visuelles, Bewegung, Kommunikation, Kombination) und erweitert durch seine Modularität ein Autorentool. Die Module der einzelnen Schritte werden gemeinsam mit Medizindidaktik-Experten spezifiziert und gestaltet.

**Ausblick:**

Um eine ständige Verbesserung der Benutzererfahrung, des Realismus und der medizinischen Validität zu gewährleisten, werden die Autoren regelmäßige iterative Evaluierungsrunden durchführen.

Darüber hinaus wird die Integration von medical tr.AI.ning in das medizinische Curriculum eine langfristige und groß angelegte Ermittlung der Vorteile und Grenzen dieses Ansatzes ermöglichen und verbesserte alternative Lehrparadigmen für die VR-Technologie bieten.

## 1. Background

Medical tr.AI.ning is an interdisciplinary joint-project funded by the Federal Ministry of Education and Research (BMBF) that aims to develop a virtual reality (VR) training platform to enable medical students to practice clinical decision-making in customizable scenarios in a safe, immersive virtual environment. Thanks to most recent advances in Artificial intelligence (AI) technology it will be possible to create realistic and holistic clinical cases with interactive, intelligent virtual patients. The medical tr.AI.ning project is motivated by the increasing focus on clinical reasoning in learning goals of medical curricula. 

## 2. Reason and project goal

By the end of 2017, the Federal Ministry of Education and Research (BMBF), the Federal Ministry of Health (BMG), the Conference of Ministers of Education (KMK) and the Conference of Health Ministers (GMK) agreed on the “Masterplan for medical schools 2020” for Germany [1]. With this decision, an enhancement of competence-based concepts for education to gain medical experts is envisaged. A main step of this process is the new catalog of learning goals (NKLM 2.0) [https://nklm.de/zend/objective/list/orderBy/@objectivePosition/studiengang/Arztrollen] being part of the new medical approbation regulations as of 2025 [2]. In contrast to the previous curriculum, this catalog places particular emphasis on social skills training.

Medical students need to be prepared for various situations in clinical decision-making that cannot be systematically trained with real patients without being burdensome for them or the students. Until now, clinical experience is often acquired through shadowing only. Unfortunately, this misses a first-person perspective and experience [3]. To target this problem, there is ongoing research for situated learning [4] with the aim of knowledge acquisition in context-based interpersonal interactions. So far, this has been successfully achieved by actor-based simulations, as it is practiced in the programs of the Medical Faculties of Münster [5], [6] and Homburg [7]. Yet, despite great acting performances and authentic conversation training, there are still system-related limitations inherent to this approach. For example, physical parameters such as various symptoms, appearance (skin color, weight, …) and age cannot be displayed authentically or varied easily. Intimate examinations represent another aspect of medical education, which cannot be trained with actors. 

The mentioned limitations illustrate why digital training and learning methods are increasingly used in the context of medical education [8]. 

Especially in VR-based training, high potential is found according to literature [9], [10]. Virtually generated training scenarios allow repetitive training of highly relevant skills within a protected learning environment. Current VR applications can display realistic visualizations, which provide immersive experiences that reportedly enhance clinical decision-making skills [9]. This experience roots in two phenomena that M. Slater calls “place illusion” and “plausibility illusion” which – when both occur – lead to a realistic response by the participant in virtual reality [11]. Thus, VR may provide a viable tool that can be used to approximate real-world situations that are impossible to train with current methods. 

Recent advances in the domain of AI and deep learning have rendered real-time speech recognition feasible and have enabled novel paradigms for face-to-face interaction with virtual agents. Unfortunately, in the context of medical education, AI has been so far primarily used for automatic evaluations, predictions of students’ performance, surgical training, or in internet platforms without deep integration of situated learning. 

Combining the potential of virtual reality simulations with advanced artificial intelligence technologies can create a new way of situated context-based, egocentric training for medical students. 

To achieve this goal, we are developing a modular, educative, digital training platform, called “medical tr.AI.ning”, with virtual, interactable agents. Within this scope we aim to integrate VR into courses of the medical curriculum that demand clinical decision making and that are otherwise limited, e.g., by not availability of patients with specific diseases or in the context of shameful physical examinations. This implies a focus on training practical skills. 

Thanks to advanced AI-technologies veridical simulations of typical clinical situations with virtual patients interacting with the trainee in verbal and nonverbal ways within a desired situational context and augmented with highly realistic, intelligent medical pathologies are provided. This allows students to repeatedly train diverse medical situations with different grades of complexity. 

A dedicated authoring tool enables the educators to create new, or modify existing training scenarios, add further pathologies or examination tools, and even customize the patients and their behavior. With this tool, teachers are able to assemble all characteristics of a virtual patient, environment, and scenario intuitively without the need for laborious implementation by external IT specialists. This will support the dissemination of our platform to other medical schools.

To ensure constant improvement and authentic evaluation, we perform regular iterative evaluations with pilot user-studies for each created scenario. With this strategy we guarantee validity and user friendliness throughout the development of medical tr.AI.ning.

More information about medical tr.AI.ning can be retrieved from our website [https://medical-training-project.de/] and our project video [https://www.youtube.com/watch?v=Oe0l_nDHyOY&t=2s].

## 3. Project plan

This joint project has been started and is conducted by leading institutes in computer science and medical didactics at the University of Münster (CVMLS, IfAS) and Saarland University (CHELM, UMTL), the University of Applied Sciences Münster (FHMS) and the art academy Saarbrücken (HBKsaar).

The project goals are scaled to four complementary developmental steps with corresponding scenarios (see table 1 (Tab. 1)), that can be used alone or combined and integrated early in medical curricula. Every step has its own main focus (visual, movement, communication, combination) and extends the authoring tool through its modularity. Modules of each step will be derived from concrete example scenarios specified and designed by our medical team including didactic experts (see table 1 (Tab. 1)). By successive adding of new modules, we aim to ensure both the scalability of the overall system and the stability of each individual training scenario. 

Medical tr.AI.ning requires knowledge of mainly three different disciplines: medicine-didactics, computer science, and design.

**Figure 1:**
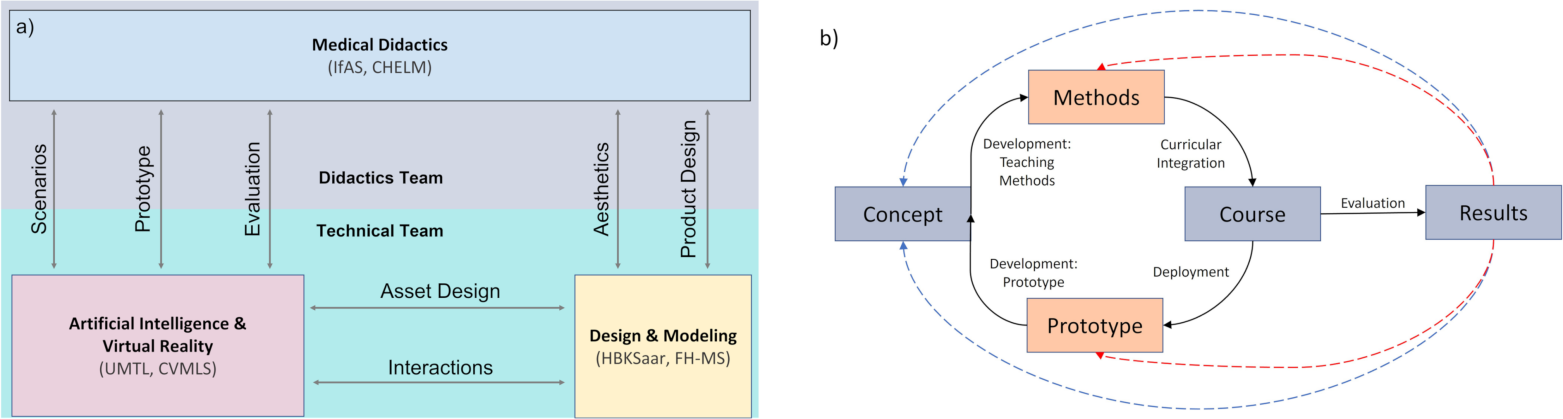
(a) Allocation of project tasks, (b) design paradigm

As illustrated in figure 1a (Fig. 1) the respective competences are divided into two broadly defined teams. The medical didactics team (IfAS, CHELM) develops teaching methods and strategies, and provides the medical background as a basis for programming and designing the scenarios in cooperation with medical specialists. The platform is implemented by the technical team (UMTL, CVMLS, FHMS). The design teams of University of Applied Sciences Münster (FHMS) and the art academy Saarbrücken (HBKsaar) provide designs, models, and animations as well as interaction concepts in VR. Overall, the tasks are strictly allocated but require close cooperation, also during the process of evaluation with iterative feedback between the different stakeholders.

The platform development employs an incremental design paradigm, with an early horizontal prototyping and small implementation-validation cycles. Every iteration (cf. figure 1b (Fig. 1))focuses on a particular training scenario and starts with a conception by all partners, where trainable medical competences, technical requirements and feasibility are evaluated and harmonized. After the conception, the medical-didactics team develops the teaching methods and courses surrounding the seamless integration of the platform, while the technical and design teams build the required system components. The results of these developments flow into dedicated educational courses with a focus on clinical reasoning that will be integrated into the medical curricula of the University of Münster and the Saarland University. These courses form the primary testbed of the platform, and the results from the evaluations within the course are used to guide the conceptual design and development in the next iteration step. 

This method allows focused vertical prototyping of each planned aspect of the platform, and early integration in medical schools, which substantially raises the probability of success and adaptability of medical tr.AI.ning. Apart from their inherent nature of needing clinical decision-making skills, according to table 1 (Tab. 1) the primary courses are chosen based on their suitability with regard to the current developmental steps. For example, the first scenario with a focus on visual findings will be implemented into the practical courses of dermatology. 

## 4. Evaluation

### 4.1. Continuous evaluation and validation process on overall user experience 

For each implemented and usable scenario, an elective with at least fifteen medical students will be initiated as a pilot-study. According to our developmental steps, they primarily test the platform with a focus on visual diagnosis. Every student is accompanied by a medical didactics expert (MDE). In the test scenario, the students are asked to speak out their thoughts related to the platform (thinking aloud-method), which will be transcribed by the MDE. To avoid any bias, students are not allowed to talk to others until the evaluation is completed.

In a second step, all students get a standardized questionnaire covering the following topics: user friendliness (intuitiveness), design, realism, learning effect, fun factor as well as the degree of being able to stick to the scenario (level of concentration). Free text is provided to give recommendations. With this method we ensure regular improvements of our platform in an iterative process based upon first-hand requirements of medical students. Continuous evaluation is done with different scenarios in all four developmental steps.

#### 4.2. Evaluation of validity and competence acquisition 

As our focus is on training clinical competences rather than to acquire theoretical knowledge, we decided to use the EPA-Concept (Entrustable Professional Activities) of Ole ten Cate [12]. This concept is based on the assumption that trust and permission to act in a clinical context can be evaluated through observation by medical experts. EPAs are assessed for 13 different competencies on a scale from 0 to 5 (“the student has no benefit from observing” to “the student is consolidated enough to guide other learners”). The assessment is based on structured requirement catalogs, but not on a fixed check-list catalog so as OSCE assessments do. This strategy emphasizes an aspired congruence between self-assessment and external assessment as well as personalized feedback that guarantee both patients’ safety and self-efficacy. 

To address competence effectiveness, two groups of students will be compared (A+VR): Group A will interact with actors while group VR will use the medical tr.AI.ning platform in similar testing scenarios. The participants of each cohort will be matched according to their medical knowledge. The scenario focuses on clinical processes in patient management. Therefore, no theoretical background like epidemiology, classification or prognosis will be addressed. The goal is to guide the student to decision making by a stepwise approach to learn how to find a correct diagnosis. During performance the students’ behavior and strategy will be evaluated in both groups by an MDE with respect to the EPA concept for comparison. 

Limitations of this study setting are mainly linked to small group sizes and different experience with VR and technical gadgets.

## 5. Perspective

Medical tr.AI.ning will be integrated into the current curricula of the involved universities at Homburg and Münster, and further universities will be invited. Extensive documentation and the development of guidelines and recommendations ensure the utilization and adaptability of our platform even outside of our project.

The authoring tool will be able to empower further experts in medical didactics to develop new scenarios and thus guarantee sustainability of medical tr.AI.ning.

## First authorship

Marvin Mergen and Anna Junga share the first authorship.

## Funding

This project received funding from BMBF (Bundesministerium für Bildung und Forschung) under grant agreement number: 16DHBKI080. We acknowledge support from the Open Access Publication Fund of the University of Muenster.

## Acknowledgements

Many thanks to Corbin Sassen, Henriette Schulze and Leon Pielage for contributing to the concept, recording and post-production of the project video.

## The medical tr.AI.ning consortium

- **University of Münster (CVMLS, IfAS)**
  Benjamin Risse, Pascal Kockwelp, Leon Pielage, Valentin Brosch, Bernhard Marschall, Anna Junga, Henriette Schulze, Ole Hätscher, Niklas Tiefenbach
- **University of Applied Sciences Münster (FHMS)**
  Tina Glückselig, Kathrin Ungru, Philipp Bozdere, Julia Leuer
- **Saarland University (UMTL, CHELM)**
  Antonio Krüger, Dimitar Valkov, Tim Düwel, André Zenner, Florian Daiber, Erum Manzoor, Norbert Graf, Marvin Mergen, Marcel Meyerheim
- **University of Fine Art Saar (HBKsaar)**
  Michael Schmitz, Mert Akbal, Corbin Sassen

## Competing interests

The authors declare that they have no competing interests. 

## Figures and Tables

**Table 1:**
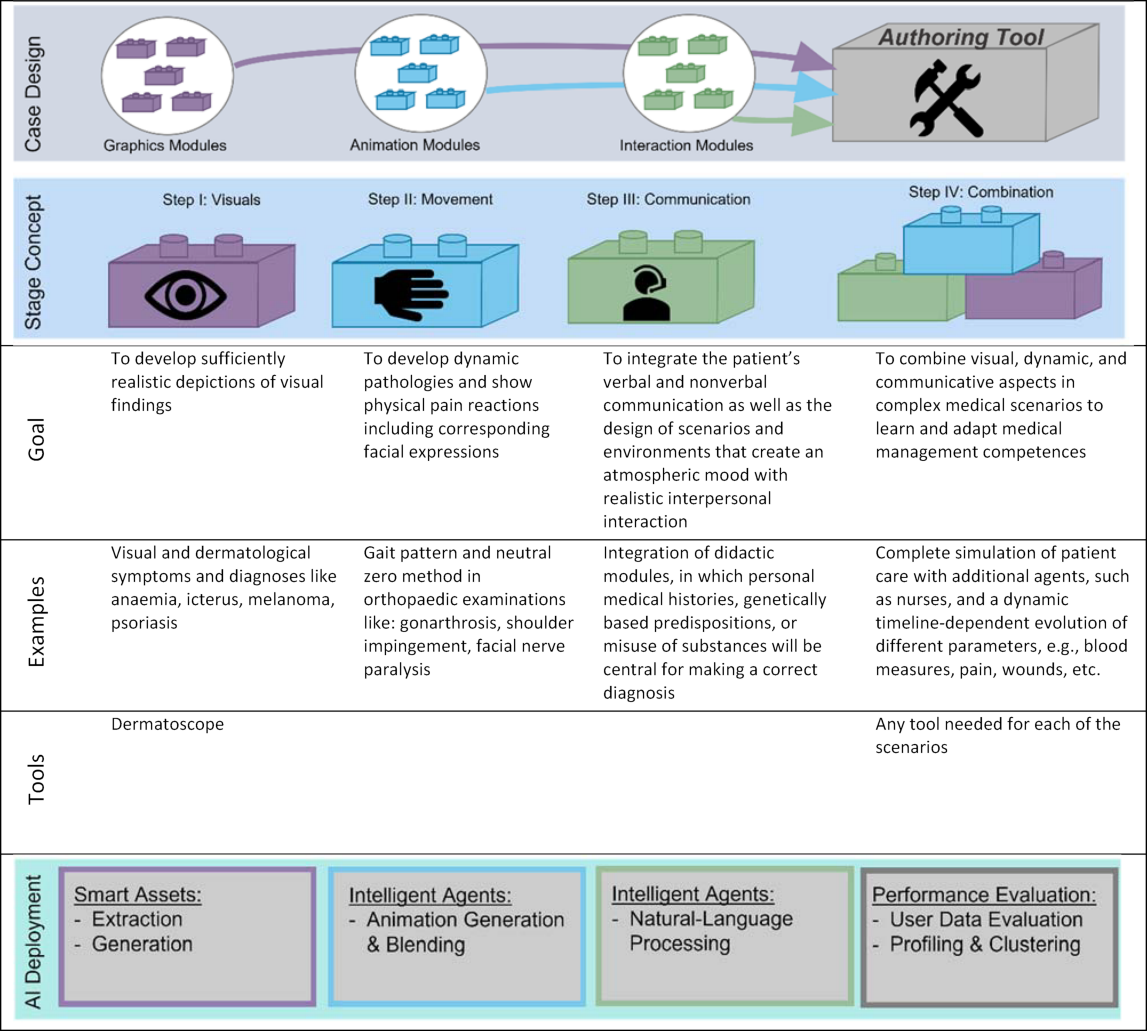
Developmental steps of the medical tr.AI.ning platform
